# INTEGRATING GEROPSYCHOLOGY SERVICES INTO MEDICAL SETTINGS

**DOI:** 10.1093/geroni/igad104.2440

**Published:** 2023-12-21

**Authors:** Ann Pearman

**Affiliations:** MetroHealth Medical System, Cleveland, Ohio, United States

## Abstract

Integrating behavioral health into medical settings, such as primary care clinics, has been shown to have a positive impact on the well-being and health of patients. Increased access to mental health services as well as increased identification of psychological issues is an important goal for all patients but may be of particular importance for geriatric patients who often have multiple chronic conditions along with an increased risk of neurocognitive disorders. This paper is intended to describe the process of starting an integrated geriatric behavioral health service line into geriatric and internal medicine clinics at a large urban hospital that serves a primarily underserved population of patients. MetroHealth Medical Centers serves over 300,000 patients, 75% of whom are uninsured or covered exclusively by Medicare and/or Medicaid. The system has one centralized geriatric medicine clinic as well as geriatric providers throughout the system. In 2021, a geropsychologist was brought on board to create a geriatric mental health service for the system. Three different models of integrated care are being used – within-clinic collaborative care, co-location of services, and referrals – and will be described further. There have been over 1200 referrals to the service since its inception, many of whom are receiving mental health care for the first time. This presentation will highlight the types of patients being referred and the efforts put forth to reach as many patients as possible. Finally, the development of Geropsychology-specific training opportunities for psychology and medical residents and fellows will be described.

